# Metformin *I*ncreases *S*arcolemma *I*ntegrity and *A*meliorates *N*euromuscular *D*eficits in a *M*urine *M*odel of Duchenne *M*uscular *D*ystrophy

**DOI:** 10.3389/fphys.2021.642908

**Published:** 2021-05-03

**Authors:** Xia Dong, Tiankun Hui, Jie Chen, Zheng Yu, Dongyan Ren, Suqi Zou, Shunqi Wang, Erkang Fei, Huifeng Jiao, Xinsheng Lai

**Affiliations:** ^1^School of Basic Medical Sciences, Nanchang University, Nanchang, China; ^2^Laboratory of Synaptic Development and Plasticity, Institute of Life Science, Nanchang University, Nanchang, China; ^3^School of Life Sciences, Nanchang University, Nanchang, China

**Keywords:** Duchenne muscular dystrophy, metformin, AMP-activated protein kinase, skeletal muscle, neuromuscular junction

## Abstract

Duchenne muscular dystrophy (DMD) is a genetic neuromuscular disease characterized by progressive muscle weakness and wasting. Stimulation of AMP-activated protein kinase (AMPK) has been demonstrated to increase muscle function and protect muscle against damage in dystrophic mice. Metformin is a widely used anti-hyperglycemic drug and has been shown to be an indirect activator of AMPK. Based on these findings, we sought to determine the effects of metformin on neuromuscular deficits in mdx murine model of DMD. In this study, we found metformin treatment increased muscle strength accompanied by elevated twitch and tetanic force of tibialis anterior (TA) muscle in mdx mice. Immunofluorescence and electron microscopy analysis of metformin-treated mdx muscles revealed an improvement in muscle fiber membrane integrity. Electrophysiological studies showed the amplitude of miniature endplate potentials (mEPP) was increased in treated mice, indicating metformin also improved neuromuscular transmission of the mdx mice. Analysis of mRNA and protein levels from muscles of treated mice showed an upregulation of AMPK phosphorylation and dystrophin-glycoprotein complex protein expression. In conclusion, metformin can indeed improve muscle function and diminish neuromuscular deficits in mdx mice, suggesting its potential use as a therapeutic drug in DMD patients.

## Introduction

Duchenne muscular dystrophy (DMD), an X-linked recessive neuromuscular disease with an incidence of 1 in 3600–6000 boys (Emery, 1991; Bushby et al., 2010), is caused by loss-of-function mutations or deletions in gene encoding dystrophin. Dystrophin is a plasma membrane protein within the dystrophin-glycoprotein complex (DGC), which forms a bridge between the extracellular matrix and the intracellular cytoskeleton in healthy sarcolemma (Muntoni et al., 2003; Davies and Nowak, 2006; Quan and Gao, 2015). In DMD, lack of dystrophin disrupts this bridge and breaks down the membrane integrity of muscle fiber, leading to muscle wasting and degeneration (Campbell and Kahl, 1989). Mdx (X-linked muscular dystrophy) mouse, which does not express dystrophin, is a well-known mouse model for DMD with a characteristic of muscle weakness (Bulfield et al., 1984; Hoffman et al., 1987b).

AMP-activated protein kinase is a serine/threonine protein kinase and a critical regulator of energy metabolism. It is activated by elevations in cellular ADP and AMP/ATP ratio due to metabolic stress, such as glucose deficiency, hypoxia, and muscle contractile activity (Winder and Hardie, 1996; Salt et al., 1998; Marsin et al., 2000). AMPK activation has been shown to play an essential role in maintaining and remodeling skeletal muscle function (Mounier et al., 2015; Kjøbsted et al., 2018). Muscle-specific AMPK β1β2 double-knockout mice display a significant myopathy with increased split, necrotic myofibers and centrally nucleated myofibers (Thomas et al., 2014). During muscle regeneration, AMPK is critical for the normal process of macrophage skewing, which is required for proper regeneration (Mounier et al., 2013). Chronic activation of AMPK reduces skeletal muscle fragility but enhances the enhances the myogenesis of slow-twitch, oxidative muscle (Ljubicic et al., 2011). In mdx mice, chronic activation of AMPK with AMPK activator AICAR (5-aminoimidazole-4-carboxamide-1-β-D-ribofuranoside) induced a slow oxidative muscle fiber program and improved the dystrophic pathology (Ljubicic et al., 2012; Ljubicic and Jasmin, 2013; Lynch, 2017). These evidences indicate that AMPK activation plays a beneficial role in skeletal muscle function maintenance and the potential therapeutic effect on DMD.

Metformin is a widely used anti-diabetic agent recommended as a first-line oral therapy for type 2 diabetes (T2D) (Inzucchi et al., 2012). Beyond the T2D, metformin has also been shown to be effective for cancer treatment, cardiovascular, anxiety, polycystic ovary syndrome, and other diseases (Barbieri, 2003; Cabreiro et al., 2013; Hong et al., 2013; Bailey, 2017; Zemdegs et al., 2019). Moreover, metformin is an indirect agonist of AMPK by targeting hepatocyte mitochondria, leading to decreased ATP/AMP ratio (Zhou et al., 2001). Considering the beneficial role of AMPK activation in skeletal muscle function, we hypothesized that metformin has a potential therapeutic effect on DMD (Viollet et al., 2012). Recently, some reports support our hypothesis. Metformin protects skeletal muscle from cardiotoxin-induced injury (Langone et al., 2014). Expression of utrophin A, which is autosomal homolog of dystrophin and serves as a compensate functionally target for the lack of dystrophin, increased after 42 days of muscle treatment with metformin in mdx mice (Ljubicic and Jasmin, 2015). More importantly, treatment with L-arginine and metformin clinically delayed the disease progression and effectively prevent the loss of DMD motor function and muscle degeneration in 7–10 years old DMD boys (ClinicalTrials.gov identifier: NCT01995032) (Hafner et al., 2016, 2019).

Here, we studied the effect of metformin on skeletal muscle and neuromuscular junction (NMJ) deficits in mdx mice. We found that metformin increased muscle strength, improved muscle sarcolemma integrity and NMJ transmission in mdx mice. Our results explored the pharmacological action of metformin on dystrophic muscles suggested the potential efficacy of metformin as a metabolic enhancer in DMD treatment.

## Materials and Methods

### Mouse Lines and Drug Treatment Dose

**FIGURE 1:**
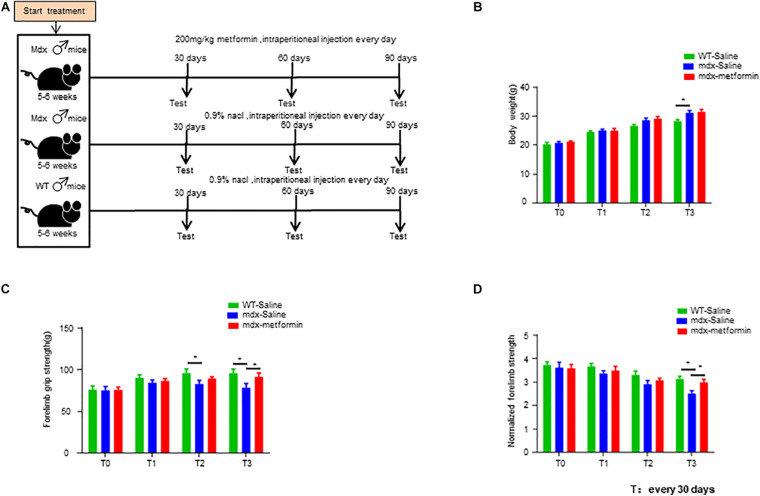
Metformin treatment increased muscle strength in mdx mice. **(A)** Scheme of the metformin treatment in mice. Intraperitoneal injection of metformin (50 μl, 200 mg/kg) or normal saline (50 μl, 0.9% NaCl) for 1–3 months. **(B)** Bodyweight of wild-type (WT) mice and metformin treated/untreated mdx mice at the beginning of treatment (T0) and after 1, 2, and 3 months (T1, T2, T3). Weight was recorded every month. Statistical analysis was conducted using a unpaired *t*-test. ^∗^*p* < 0.05, ^∗∗^*p* < 0.01. *N* = 6 mice per group. **(C)** Forelimb grip strength of WT mice and metformin treated/untreated mdx mice at the beginning of treatment (T0) and after 1, 2, and 3 months (T1, T2, T3). Forelimb grip strength was recorded once a month. Statistical analysis was conducted using a unpaired *t*-test. ^∗^*p* < 0.05, ^∗∗^*p* < 0.01, *N* = 6 mice per group. **(D)** Normalization of forelimb grip strength by body weight at the beginning of treatment (T0) and at 1, 2, and 3 months after treatment (T1, T2, T3) in WT and metformin treated/untreated mdx mice. Statistical analysis was conducted using a unpaired *t*-test. ^∗^*p* < 0.05, ^∗∗^*p* < 0.01, ^∗∗∗^*p* < 0.001. *N* = 6 mice per group.

Mdx mice (C57BL/10ScSn-Dmd^mdx^/J) were purchased from The Jackson Laboratory (stock # 001801). Before treatment, a total of 50 male mdx mice and 20 male wild-type control mice (WT, C57BL/10ScSn/J) aged 4–5 weeks were acclimatized for 1 week in the animal facility before the experimental procedures. For treatment, mdx mice were randomly assigned to two subgroups. One group was intraperitoneally injected with metformin (D150959-5G, Sigma, 200 mg/kg, *n* = 25) everyday, and the other group was intraperitoneally injected with normal saline (0.9% NaCl, *n* = 25) everyday. Wild-type mice (*n* = 20), were intraperitoneally injected with normal saline (0.9% NaCl) everyday. After 30, 60, and 90 days of treatment, six mice from each group were taken out for further experiments (Figure 1A). Experimental procedures were approved by the Institutional Animal Care and Use Committee of the Nanchang University.

### Reagents and Antibodies

Chemicals were purchased from Sigma-Aldrich unless otherwise indicated. CF568 α-bungarotoxin (α-BTX, #00006, 1:1000 for staining) was purchased from Biotium. Antibodies including AMPKα (2603, 1:1000 for Western blot), phospho-AMPKα (Thr^172^) (2535, 1:1000 for Western blot), Neurofilament (2837S, 1:1000 for staining) and Synapsin (5297S, 1:1000 for staining) were from Cell Signaling Technology; laminin (#041M4799, 1:200 for staining) from Sigma-Aldrich; GAPDH (HRP-60004; 1:2000 for Western blot) and α-dystroglycan (106110; 1:1000 for Western blot and staining) from Abcam, eMyHC (F1.652; 1:200 for staining) from DSHB. Alex-aFluor-488 goat anti-rabbit IgG (A11034, 1:1000 for staining), AlexaFluor-488 goat anti-mouse IgG (A11029, 1:1000 for staining), and AlexaFluor-568 goat anti-rabbit IgG (A11036,1:1000 for staining) were from Invitrogen. HRP-conjugated goat anti-rabbit IgG (32260), goat anti-mouse IgG (32230) antibodies (1:2000 for Western blot) were from Invitrogen.

### Body Weight and Muscle Strength

Body weight was measured by the electronic scale. Muscle strength was measured by SR-1 hanging scale (American Weigh Scales) (Shen et al., 2013; Barik et al., 2014). Briefly, mice were allowed with forelimbs to grasp a grid that was connected to a scale. Their tails were gently pulled until the grid was released by the forelimbs, and readings of the scale were recorded.

### *In vivo* Twitch and Tetanic Force Measurement

Torque muscle tension analysis was performed on male mice as previously reported (Ingalls et al., 1985; Arpke et al., 2013). Briefly, mice were anesthetized with isoflurane continuously supplied by VetFlo anesthesia system (Kent Scientific) and placed on a 37°C heating pad. With gentle pressing the knee clamps, left feet were fixed onto the footplate that was connected to the servomotor (Aurora Scientific 1300A). For muscle stimulation, two needle electrodes were inserted subcutaneously into TA muscle close to the knee. The best position of muscle contraction was found by adjusting the distance between the footplate and the knee and measuring the muscle force by stimulating muscle with a single electrical stimulation (100 mA current, 0.2 ms pulse width). When the muscle force was no longer increasing, the position was the best position of muscle contraction. To identify best stimulation strength, a single muscle electrical stimulation was given starting at 100 mA current, 0.2 ms pulse width in the best position, the muscle force was measured every 30 mA increase, with an interval of 30 s. When the muscle force was no longer increasing, the current was the best stimulation strength. In the best position and the best stimulation strength, twitch force was measured by stimulating muscle with a single electrical stimulation (0.2 ms pulse width), repeating 10 times with an interval of 30 s. Tetanic force was measured by stimulating muscle with 300 ms duration, 0.2 ms pulse width at a series of frequencies from 50 to 150 Hz (50, 100, 150 Hz) with an interval of 2 min. For nerve stimulation, the sciatic nerve was exposed at thigh level and stimulated by two needle electrodes that were close to both sides of the nerve. After finding out the best position and the best stimulation intensity, nerve-stimulated twitch and tetanic were measured. Twitch and tetanic forces were normalized by body weight.

### Electromyography and Electrophysiological Recording

Electrophysiology recording was performed as described previously (Barik et al., 2016; Zhao et al., 2017). Mice were anesthetized with ketamine and xylazine mixture (100 and 10 mg/kg body weight, respectively) on a 37°C heating pad, left hemi-diaphragm together with ribs and phrenic nerves were dissected, mounted on Sylgard gel, and perfused in oxygenated (95% O_2_/5% CO_2_) Ringer’s solution (136.8 mM NaCl, 5 mM KCl, 12 mM NaHCO_3_, 1 mM NaH_2_PO_4_, 1 mM MgCl_2_, 2 mM CaCl_2_, and 11 mM D-glucose, pH 7.3) at room temperature. To record miniature endplate potentials (mEPPs), microelectrodes with 20–50 MΩ were filled with 3M KCl and pierced into the muscle fiber with the macroscopic adjacent of nerve with the resting potential between –65 and –80 mV. Ten recordings were performed and each last about 3 min. For endplate potentials (EPPs), phrenic nerves were stimulated by a suction electrode with suprathreshold square pulses. Muscle contraction was blocked by adding 2.5 μM μ-Conotoxin. Twenty minutes later the phrenic nerve was sucked into electrode and stimulated. The data was collected with MultiClamp 700B amplifier which digitized (10 kHz low-pass filtered) with Digidata 1550A and analyzed in Clampfit10.5 software.

### Serum Creatine Kinase Measurement

Mice were deeply anesthetized with isoflurane, and blood samples were obtained by cardiac puncture. Blood was centrifuged at 10,000 rpm for 10 min at 4°C. Serum creatine kinase (CK) levels were measured using a CK activity assay kit (MAK116-1KT, Sigma) and a spectrophotometer according to the manufacturer’s protocol. When measuring CK, the wavelength of the instrument was set to 340 nm.

### Immunofluorescence

Muscles were fixed with 4% PFA in PBS at room temperature 20 min, rinsed with 0.1 M glycine in PBS for 30 min, and incubated with the blocking buffer (5% BSA, 2% Triton X-100, 5% goat serum in PBS) for 2 h at room temperature. They were then incubated with primary antibodies in blocking buffer at 4°C overnight. After washing three times for 1 h each with 2% Triton X-100 in PBS, the samples were incubated with fluorescent-labeled secondary antibodies in PBS 2 h at room temperature. Muscle samples were then washed with 2% TritonX-100 in PBS three times for 1 h each and mounted with Vectashield mounting medium (H1200) and coverslip. For muscle cross-section staining, muscles were fixed with 4% PFA in PBS at 4°C overnight. After dehydration by 30% sucrose at 4°C overnight, muscles were frozen at –80°C in Cryo-embedding medium (Ted Pella) and cut into 15 μm sections on a Frozen slicing machine (Leica CM900). Sections were incubated with the blocking buffer for 2 h at room temperature and then with primary antibodies in the blocking buffer at 4°C overnight. After washing three times for 30 min each with 2% Triton X-100 in PBS at room temperature, the samples were incubated with fluorescent-labeled secondary antibodies in PBS 2 h at room temperature. The samples were mounted with Vectashield mounting medium. Z serial images were collected with a Zeiss confocal laser scanning microscope (LSM 700) and collapsed into a single image.

### Hematoxylin and Eosin Staining (HE)

Muscle cross sections (15 μm) were stained with hematoxylin and eosin, dehydrated through ascending, graded ethanol washes, cleared with toluene, and then mounted with Permount (Fisher Scientific, Ottawa, Canada). They were visualized at 4.2× magnification using a light microscope and the ratio of myofibers with centralized nuclei to the total number of fibers in the muscle section was counted from the acquired high resolution images.

### Evans Blue Dye Staining

Evans blue (EBD) were purchased from Sigma (St. Louis, MO, United States). The dyes were dissolved in phosphate-buffered saline (PBS; 0.15M NaCl, 10 mM phosphate buffer, pH7.0) sterilized by passage through membrane filters with a pore size of 0.2 μm and kept at 4°C. Dye solution was injected intravenously through the celiac vein (1 mg dye/0.1 ml/10 g body weight) and 3–24 h after injection, mice were killed with an over-dose of ether gas. To prepare cryosections, fresh tissues were embedded in OTC compound (Miles, Elkhart, IN, United States) and frozen immediately in isopentane at –70°C. Frozen tissues were then sectioned at a thickness of 15 μm by a cryostat. EBD binds to albumin and is detected by fluorescence microscopy (at 568 nm) in the extracellular space. Presence of the proteinbound dye inside the muscle fiber indicates damage to the sarcolemma. Here, the sections were assessed blindly, and the myofibers were judged in a binary fashion, as positive or negative for intracellular EBD.

### Masson’s Trichrome Staining

The masson’s trichrome staining was conducted using ready-to-use kit (Masson’s Trichrome Stain Kit, G1340, Solarbio). Briefly, the muscle tissue cut into 10 μm sections on a Frozen slicing machine (Leica CM900). Sections were then immersed in Bouin’s solution (G2331, Solarbio) at room temperature overnight; stained in Weigert’s hematoxylin for 5 min, differentiation in acid ethanol solution for 10 s, washing with tap water for 1 min, then used Masson blue solution to return to blue for 5 min and washed again with tap water for 1 min and rinsed in distilled water for 1 min. Next, the sections were stained in Ponceau Magenta staining solution for 5 min, washed with phosphomolybdic acid solution for 1 min, and washed with 0.2% acetic acid for 1 min. Dyed directly in the aniline blue staining solution for 1 min, and washed with 0.2% acetic acid for 1 min. Finally, dehydrated and mounted.

### Electron Microscopy

Precooling glutaraldehyde fixative solution before muscle tissue extraction. Removed TA muscle from the body after the mice were deeply anesthetized. the muscle tissue samples were cut into 1 mm^3^ pieces and immersed into 0.5% glutaraldehyde fixative for 2 min. Fixed in 2.5% glutaraldehyde fixative solution at 4°C overnight. Fixed tissues were washed with PBS and rinses with 100% propylene oxide three times. Muscle samples were embedded in plastic resin. Ultrathin cross sections were cut at 80∼100 nm and mounted on 200-mesh unsupported copper grids and stained with a solution containing 50% methanol, 3.5% sodium citrate, 3% uranylacetate, and 2.6% lead nitrate. Micrographs were taken by using JEOL 100CXII operated at 80 KeV.

### Western Blot Analysis

Western blot was performed as described previously (Barik et al., 2014). Briefly, each protein sample was separated by SDS-polyacrylamide gel electrophoresis, transferred to a nitrocellulose membrane, blocked with 5% milk in Tris-buffered saline/0.1% Tween 20, and hybridized with the following primary antibodies: anti-α-dystroglycan (1:1000, Abcam), anti-AMPKα (1:1000, Cell Signaling), anti-Phospho-AMPKα (Thr172) (1:1000, Cell Signaling), and GAPDH (1:2000, Abcam). The membranes were then incubated with anti-rabbit or anti-mouse horseradish peroxidase (HRP)-conjugated secondary antibody (1:2000, Invitrogen). Detection of the signal was accomplished using western HRP chemiluminescence (ECL) reagents (Thermo), and imaging of the blots was performed using ChemiDoc^TM^ MP System (Bio-Rad). To analyze the blots, Image Lab^TM^ Software (Bio-Rad) was used to quantify band intensity and calculate the absorbance ratio of the target protein to the loading control.

### Quantitative Real-Time PCR Analysis

Total RNA was purified from gastrocnemius with Trizol reagent (Invitrogen, NY, United States), 500 ng of total RNA were converted to cDNA by using a reverse transcription kit (Takara, Japan) and oligo (dT) primers. cDNAs were used as template in qPCR in a 20 μL reaction system containing SYBR GreenER qPCR mix with gene-specific primers (Table 1). PCR included an initial step at 95°C (3 min), followed by 40 cycles consisting of denaturation at 95°C (15 s), annealing and extension at 60°C (60 s). Using this method, the GAPDH were used as reference in each sample.

**TABLE 1:** Primer sequence used in the qRT-PCR.

**Name**	**Forward (5′-3′)**	**Reverse (5′-3′)**
*GAPDH*	GTGAAGGTCGGTGTGAACGG	CAAGCTTCCCATTCTCGGCCT
*Dystrobrevin*	CGGCTTGATGAAGAACACAGGC	CGATGGTGAAGGAGATGTCAGG
*β1-syntrophin*	AGCCTCTGTCATCCCAGTCCTT	GTGTGCTTAGCATCTGGCGAGT
*β2-syntrophin*	GCTGTGACTGAGAAGGACTTGC	GTGACCGACATCCAGAACCTGA
*α-syntrophin*	CAGTTGGTGGATGGCTGTCATC	GTGAAGCCCTTGTCGATGTGCA
*Dystroglycan*	GTGGTTGGCATTCCAGACGGTA	CAGTGTAGCCAAGACGGTAAGG
*Sarcoglycan α*	GGTCGTGTGTTTGTGCATCC	CACGATCTTCTGGAGTGGGG
*Sarcoglycan β*	GGCAACTTAGCCATCTGCGTGA	GTGGAACTCCATGCTATCACACC
*Sarcoglycan γ*	GTGACAGTCAGTGCTCGCAACT	GCAGAGAACAGTGGCTTGCCAT
*Sarcoglycan δ*	TGAGACTGGAGTCCAAGGATGG	CTCGAAGACCTTCTGCCTCGTT

### Statistical Analysis

Data were analyzed by unpaired *t*-test, one-way ANOVA, and two-way ANOVA. Unless otherwise indicated, data were shown as mean ± SEM. Statistical difference was considered when *p* < 0.05.

## Results

### Increased Muscle Strength After Metformin Treatment in mdx Mice

The dose of metformin whas chosen based on previous studies (Mantuano et al., 2018). Here, we opted for intraperitoneal injection for a simpler determination of the daily dose to be administered to each mouse. Male mdx and WT mice aged 5–6 weeks were intraperitoneally injected with metformin (50 μl, 200 mg/kg) or normal saline (50 μl, 0.9% NaCl) for 1–3 months (Figure 1A). No differences in bodyweight were found between metformin treated and control group (Figure 1B). However, the forelimb grip strength of metformin-treated mdx mice was significantly increased by 8.54 ± 2.733% after 3 months of treatment (T3) (Figures 1C,D).

**FIGURE 2:**
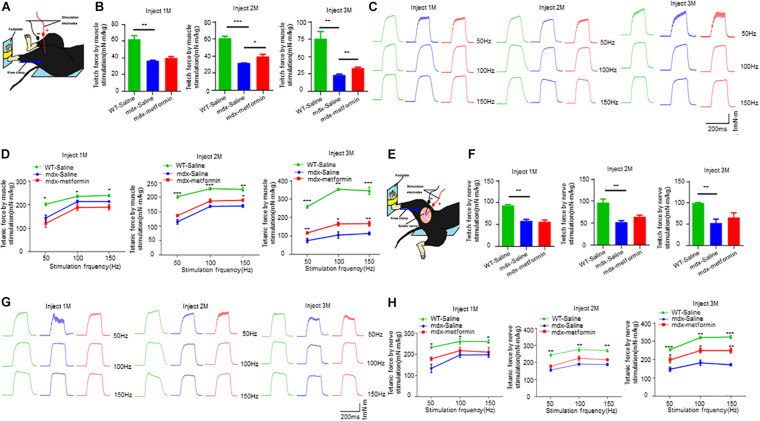
Metformin increased twitch and tetanic force by nerve and muscle stimulation in mdx mice. **(A)** Scheme of in vivo muscle twitch and tetanic force measurement by muscle stimulation. **(B)** After 1, 2, and 3 months of treatment, twitch force between WT mice and metformin treated/untreated mdx mice by muscle stimulation. After 2 and 3 months of treatment with metformin, the twitch force of mdx mice increased. Statistical analysis was conducted using a one-way ANOVA. *p < 0.05, **p < 0.01, ***p < 0.001. *N* = 6 mice per group. **(C)** After 1, 2, and 3 months of treatment, representative tetanic curves at stimulation frequency 50, 100, and 150 Hz by muscle stimulation. **(D)** After 1, 2, and 3 months of treatment, tetanic force between WT mice and metformin treated/untreated mdx mice by muscle stimulation. After treatment with metformin for 3 months, the tetanic force of mdx mice increased. Statistical analysis was conducted using a two-way ANOVA. *p < 0.05, **p < 0.01, ***p < 0.001. *N* = 6 mice per group. **(E)** Scheme of *in vivo* muscle twitch and tetanic force measurement by nerve stimulation. **(F)** After 1, 2, and 3 months of treatment, twitch force between WT mice and metformin treated/untreated mdx mice by nerve stimulation. Statistical analysis was conducted using a one-way ANOVA. **p < 0.01. *N* = 6 mice per group. **(G)** After 1, 2, and 3 months of treatment, representative tetanic curves at stimulation frequency 50, 100, and 150 Hz by nerve stimulation. **(H)** After 1, 2, and 3 months of treatment, tetanic force between WT mice and metformin treated/untreated mdx mice by nerve stimulation. After treatment with metformin for 3 months, the tetanic force of mdx mice increased. Statistical analysis was conducted using a two-way ANOVA. *p < 0.05, **p < 0.01, ***p < 0.001. *N* = 6 mice per group.

To determine the pathological mechanism of metformin increasing muscle strength, we measured muscle contractions by stimulating nerves and muscles, respectively. Nerve stimulation requires appropriate neuromuscular conduction to cause muscle contraction, whereas direct muscle stimulation does not. As shown in Figures 2A–D, by direct muscle stimulation, twitch and tetanic force were increased after metformin treatment in mdx mice, even at 2 months after treatment, earlier than forelimb grip strength increase. However, by nerve stimulation, there was no difference of twitch force between untreated and treated mdx mice, only tetanic force increased in treated mdx mice after 3 months (Figures 2E–H). Together, these results suggest that muscle strength improvement in mdx mice by metformin treatment may be mainly due to repaired muscle membrane, because twitch and tetanic force increased more and earlier by direct muscle stimulation.

### Improved the Sarcolemma Integrity of Muscle Fibers

**FIGURE 3:**
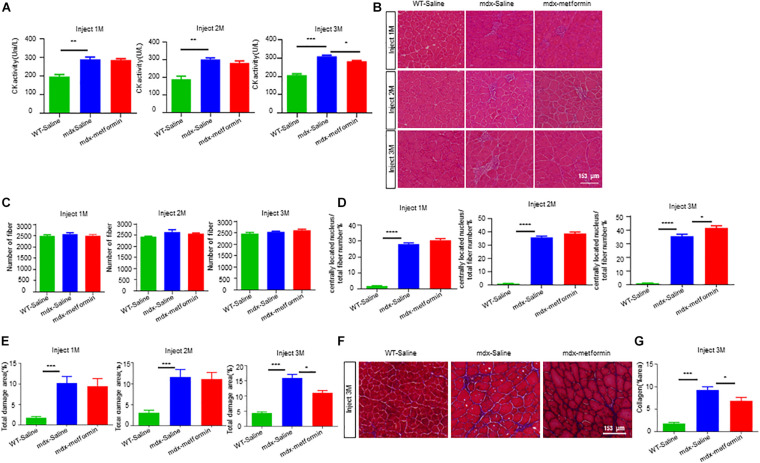
Metformin promoted muscle regeneration in mdx mice. **(A)** Plasma creatine kinase levels in WT mice and metformin untreated/treated mdx mice after 1, 2, and 3 months. Statistical analysis was conducted using a unpaired *t*-test. ^∗^*p* < 0.05, ^∗∗^*p* < 0.01, ^∗∗∗^*p* < 0.001, ^*⁣*⁣**^p < 0.0001. *N* = 7 mice per group. **(B)** Representative images of H&E staining on TA muscle sections of WT mice and metformin untreated/treated mdx mice 1, 2, and 3 months. Scale bar, 153 μm. **(C)** Quantitative analysis of the total number of muscle fibers of TA muscle after 1, 2, and 3 months of WT mice and metformin untreated/treated mdx mice. Statistical analysis was conducted using a unpaired *t*-test. *N* = 6 mice per group. **(D)** Ratio of muscle fibers with central nucleation to total number of muscle fibers. Statistical analysis was conducted using a unpaired *t*-test. ^∗^*p* < 0.05, ^∗∗^*p* < 0.01, ^∗∗∗^*p* < 0.001. *N* = 6 mice per group. **(E)** Percentage of total area of damage area in TA muscles from WT and metformin untreated/treated mdx mice groups. Statistical analysis was conducted using a unpaired *t*-test. ^∗^*p* < 0.05, ^∗∗∗^*p* < 0.001. *N* = 6 mice per group. **(F)** Representative Masson’s trichrome staining images of the TA muscles from WT mice, metformin untreated/treated mdx mice for 3 months (collagen fibers were stained blue and muscle red). Scale bar, 153 μm. **(G)** Quantification of the percentage of collagen fiber area in TA muscle after 3 months in WT mice and metformin untreated/treated mdx mice. Statistical analysis was conducted using a unpaired *t*-test. ^∗^*p* < 0.05, ^∗∗∗^*p* < 0.001. *N* = 6 mice per group.

In DMD patients and mdx mice, loss of dystrophin leads to muscle sarcolemma fragile due to the disruption of sarcolemma integrity (Campbell and Kahl, 1989; Quan and Gao, 2015). We then asked whether metformin improves the muscle sarcolemma integrity in mdx mice. Firstly, we analyzed the serum levels of CK, a skeletal muscle enzyme released during fiber degeneration. As shown in Figure 2. A, CK levels of mdx mice was expectedly higher than WT mice, but significantly decreased after 3-month metformin treatment (310.2 ± 5.757 vs. 283.2 ± 2.926), suggesting a decrease in severity of muscle damage. Furthermore, tibialis anterior (TA) muscles were stained with HE and analyzed for the presence of centrally located nuclei (CLN). A 6.136 ± 2.124% increase in CLN percentage was observed in the metformin-treated mdx TA muscle compared with control, and the muscle total damage area (necrosis, infiltration, and non-muscle area) decreased by 3.393 ± 1.503%, suggesting promotion of muscle fiber regeneration after metformin treatment (Figures 3B–E). Moreover, Masson’s trichrome staining was also used to visualize the fibrosis in muscle by detecting collagen fibers. As shown in metformin treatment reduced the area ratio of muscle fibrosis in mdx mice after 3 months (Figures 3F,G).

**FIGURE 4:**
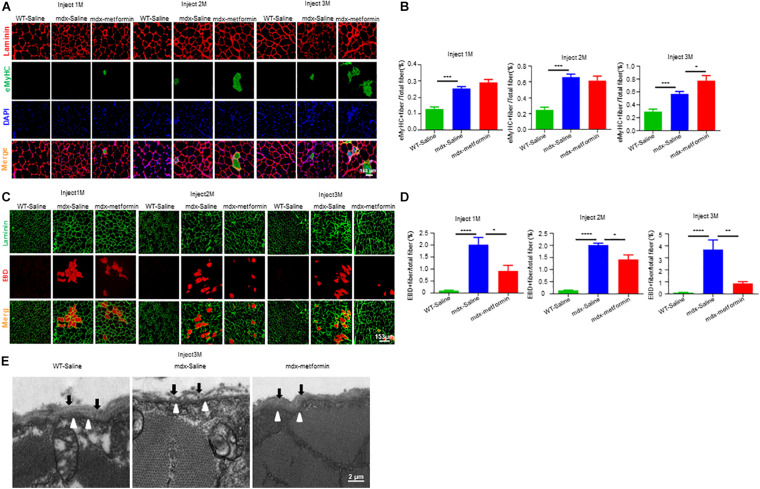
Metformin improved muscle fiber sarcolemma integrity in mdx mice. **(A)** Representative images of TA muscle in WT mice and metformin untreated/treated mdx mice for 1–3 months (eMyHC in green; Laminin in red; DAPI in blue). Scale bar, 153 μm. **(B)** Quantification of eMyHC**+** fiber in TA muscle after 1, 2, and 3 months in WT mice and metformin untreated/treated mdx mice. Statistical analysis was conducted using a unpaired *t*-test. ^∗^*p* < 0.05, ^∗∗∗^*p* < 0.001, ^*⁣*⁣**^p < 0.0001. *N* = 6 mice per group. **(C)** Representative images of Evans blue dye (EBD, red) infiltration in TA muscle after 1, 2, and 3 months in WT mice and metformin untreated/treated mdx mice (laminin in green; EBD in red). Scale bar, 153 μm. **(D)** Quantification of EBD**+** fiber in TA muscle tissue after 1, 2, and 3 months in WT mice and metformin untreated/treated mdx mice. Statistical analysis was conducted using a unpaired *t*-test. ^∗^*p* < 0.05, ^∗∗^*p* < 0.01, ^∗∗∗^*p* < 0.001. *N* = 6 mice per group. **(E)** Representative electron microscopy images of the TA muscles from WT mice, metformin untreated and treated mdx mice for 3 months. The black arrows highlight the substrate structure of the extracellular basement membrane, and the hollow arrows point to the dense plasma membrane. Scale bar, 2 μm.

To further determine the effect of metformin on muscle regeneration in mdx mice, we investigated embryonic myosin heavy chain (eMyHC) positive fibers, which represents newly formed muscle fibers, in TA muscle. Consistently, an increased number of eMyHC positive muscle fibers were observed in metformin-treated mdx mice (Figures 4A,B). To investigate if metformin can improve the integrity of the muscle membrane by reducing the fragility of the muscle fiber membrane in mdx mice, Evans Blue Dye (EBD) assay was used to evaluate the muscle membrane integrity of TA muscle fiber in mdx mice (Hamer et al., 2002). As shown in Figures 4C,D, after metformin treatment, the EBD positive muscle fibers decreased in mdx mice. These results suggest that metformin treatment improved the muscle fiber membrane integrity in mdx mice. To further confirm this, cross sections of TA muscles were collected for electron microscopy examination. As shown in Figure 4E, in metformin-treated mdx mice, the muscle basement membrane was significantly thickened and integrated more than that in control mice. Together, these results suggest that metformin treatment significantly improved muscle membrane integrity in mdx mice.

### Improved Neuromuscular Transmission

**FIGURE 5:**
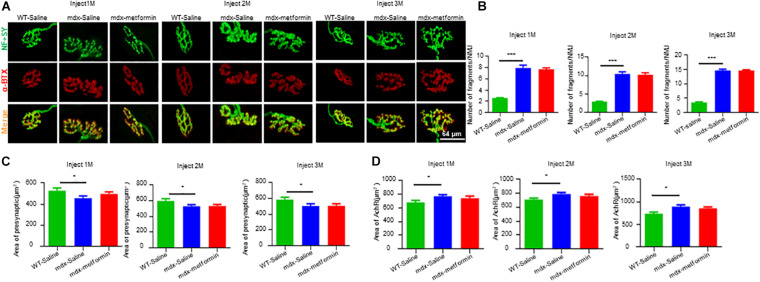
Metformin did not alter NMJ structure in mdx mice. Extensor digitorum longus (EDL) muscle was stained whole-mount with Alexa Fluor 594-conjugated-BTX (red) to label acetylcholine receptor (AChR) and antibodies against NF and SYN (NF/SYN; green) to label axon and terminals. **(A)** Representative images of NMJs after 1, 2, and 3 months in WT and metformin untreated/treated mdx mice. The Scale bar, 64 μm. **(B)** Quantitative analysis of the fragmented number of AChR clusters in EDL muscle tissues of WT mice and metformin untreated/treated mdx mice after 1, 2, and 3 months. Hundred NMJs per mouse were analyzed. Statistical analysis was conducted using a one-way ANOVA. *N* = 6 mice per group. ^∗∗∗^*p* < 0.001. **(C)** Quantitative analysis of the presynaptic area of NMJs in EDL muscle tissue after 1, 2, and 3 months of WT mice and metformin untreated/treated mdx mice. Twenty NMJs per mouse were analyzed. Statistical analysis was conducted using a one-way ANOVA. ^∗^*p* < 0.05. *N* = 6 mice per group. **(D)** Quantitative analysis of NMJs postsynaptic AChR area (AChR area) in EDL muscle tissue of WT mice and metformin untreated/treated mdx mice after 1, 2, and 3 months. Twenty NMJs were analyzed for each mouse. Statistical analysis was conducted using a one-way ANOVA. ^∗^*p* < 0.05. *N* = 6 mice per group.

Neuromuscular junction decline is a hallmark of DMD patients and mdx mice. To investigate whether metformin treatment diminishes NMJ structural deficits in mdx mice, extensor digitorum longus (EDL) muscles were stained with α-BTX to label AChR clusters and with anti- Neurofilament (NF)/ Synapsin (SYN) antibodies to label nerve terminals. As expected, NMJs in mdx mice displayed more fragments. However, no difference was observed between metformin-treated and control mdx mice, suggesting metformin treatment did not alter NMJ structure in mdx mice (Figures 5A–D).

**FIGURE 6:**
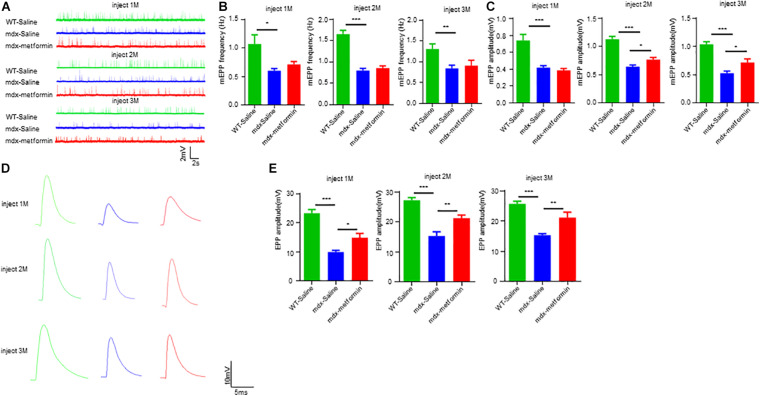
Metformin improved neuromuscular transmission in mdx mice. **(A)** Representative traces of mEPP in WT mice and metformin untreated/treated mdx mice after 1–3 months. **(B)** Quantitative analysis of mEPP frequency in WT mice and metformin untreated/treated mdx mice after 1–3 months. Statistical analysis was conducted using a one-way ANOVA. *p < 0.05, **p < 0.01, and ***p < 0.001. *N* = 6 mice per group. **(C)** Quantitative analysis of mEPP amplitude in WT mice and metformin untreated/treated MDX mice after 1–3 months. mEPP amplitude increased after metformin treatment for mdx mice at 2 and 3 months. Statistical analysis was conducted using a one-way ANOVA. *p < 0.05, **p < 0.01, ***p < 0.001. *N* = 6 mice per group. **(D)** Representative traces of EPP in WT mice and metformin untreated/treated mdx mice after 1–3 months. **(E)** Quantitative analysis of EPP amplitude in WT mice and metformin untreated/treated MDX mice after 1–3 months. EPP amplitude increased after metformin treatment for mdx mice at 2 and 3 months. Statistical analysis was conducted using a one-way ANOVA. *p < 0.05, **p < 0.01, ***p < 0.001. *N* = 6 mice per group.

Neuromuscular junction transmission is impaired in DMD patients and mdx mice with reductions in the amplitudes of mEPP and in AChR density. To test whether metformin treatment ameliorates transmission deficits in mdx mice, we recorded mEPPs and EPPs of muscles in metformin-treated and control mdx mice. mEPPs represents local depolarizations around endplates in response to spontaneous ACh release, as shown in Figures 6A–C, mEPP frequency was not altered, but amplitude was increased after 2-month metformin treatment in mdx mice. These results indicate metformin treatment does not affect the presynaptic acetylcholine release but increase the postsynaptic AChR density in mdx mice. After that, we measured EPPs, the local electrical responses in response to nerve stimulation. Accordingly, the EPP amplitude was increased after 2-month metformin treatment in mdx mice (Figures 6D,E), suggesting that metformin treatment in mdx mice improved the postsynaptic signal received from nerve stimulation. Together, our findings suggest that metformin improved neuromuscular transmission by postsynaptic, but not presynaptic, component in muscles of mdx mice.

### Upregulated AMPK Phosphorylation and DGC Proteins Expression

**FIGURE 7:**
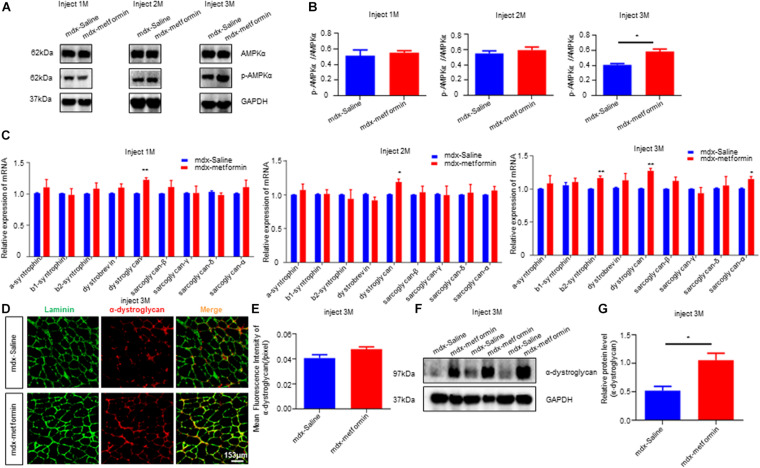
Metformin increased AMPK phosphorylation and DGC proteins expression in mdx mice. **(A)** The figure shows a representative western blot of total AMPKα and phosphorylated AMPKα (p AMPKα) from the tibialis anterior muscle (TA) of metformin treated/untreated mdx mice after 1, 2, and 3 months. *N* = 6 mice per group. **(B)** Quantification of p AMPKα/ AMPKα of **(A)**. *N* = 3 mice group. Statistical analysis was conducted using a unpaired *t*-test. *P < 0.05, *N* = 6 mice group. **(C)** Detection of mRNA levels of DGC components in metformin untreated/treated mdx mice after 1, 2, and 3 months. Membrane protein dystroglycan was upregulated after metformin treated. Statistical analysis was conducted using a unpaired *t*-test. *p < 0.05, **p < 0.01, ***p < 0.001. *N* = 6 mice per group. **(D)** Representative images of α-dystroglycan immunofluorescence of metformin untreated/treated mdx mice after 3 months. Scale bar, 153 μm. **(E)** Quantitative analysis of α-dystroglycan immunofluorescence intensity in metformin untreated/treated mdx mice after 3 months. Statistical analysis was conducted using a unpaired *t*-test. *N* = 6 mice per group. **(F)** Western blot of α-dystroglycan protein in metformin untreated/treated mdx mice after 3 months. **(G)** Quantitative analysis of the relative expression level of α-dystroglycan protein in metformin untreated/treated mdx mice after 3 months. Statistical analysis was conducted using a unpaired *t*-test. *p < 0.05. *N* = 6 mice per group.

Previous studies reported metformin indirectly stimulates AMPK signaling in mice. To validate this, we detected the ratio of phosphorylated AMPK (pAMPK)/AMPK in TA muscle samples using western blot (Figures 7A,B). Expectedly, metformin treatment induced a further increase in the pAMPK/AMPK ratio, and this ratio increased more after the 3-month treatment (T3) (Figure 7B). As the results described above, metformin treatment elevated twitch and tetanic contraction by direct muscle stimulation, reduced the muscle membrane’s fragility and improved neuromuscular transmission by postsynaptic component (Figures 2, 4, 6). These results indicate that metformin treatment may increase production of components related to muscle membrane integrity in mdx mice. To test this, we examined mRNA levels of Dystrophin-associated glycoprotein complex (DGC), the protein complex that maintains the stability and functional contraction of the muscle membrane. DGC is composed of transmembrane protein (dystroglycans, sarcoglycans) and intracellular protein (syntrophins, dystrobrevin, dystrophin). *β-syntrophin, dystroglycan and sarcoglycan-a* were upregulated after the 3-month metformin treatment in mdx mice. Notably, mRNA level of *dystroglycan* increased, even in 1-month metformin-treated mdx mice, earlier than other involved proteins (Figure 7C). Moreover, immunofluorescence imaging analysis of histological sections from metformin-treated TA muscles showed a noticeable increase in the expression of α-dystroglycan in the sarcolemma (Figures 7D,E). Consistently, western blot results also showed that α-dystroglycan protein in the muscles of mdx mice was increased after metformin treatment for 3 months (Figures 7F,G).

## Discussion

Metformin is one of the most effective and safest agents for anti-hyperglycemic and currently employed as a first-line oral therapy for T2D. It has also demonstrated additional beneficial effects on cancer, cardiovascular disorders, mental disorders, immune and other metabolic diseases (Barbieri, 2003; Hong et al., 2013; Stagi et al., 2017; Ursini et al., 2018; Zemdegs et al., 2019). Previous studies have illustrated the protective effect of metformin on skeletal muscle damage and a potential role in easing DMD patients’ symptoms (Langone et al., 2014). In this study, we investigated the effect of metformin on neuromuscular deficits in the DMD mouse model mdx. Interestingly, we found that metformin treatment alleviates dystrophin deficiency induced muscle weakness. Firstly, metformin treatment in mdx mice increased the forelimb strength force (Figure 1), consistent with previous findings that metformin increases muscle strength in the mdx mice (Mantuano et al., 2018). Secondly, after metformin treatment in mdx mice, muscle contraction was significantly increased by muscle stimulation (Figure 2). Since the core clinical symptom of DMD is progressive muscle weakness, it first affects the muscles of limb close to the trunk, followed by a gradual loss of muscle strength that usually makes the patient sit in a wheelchair before the age of 13 (Hoffman et al., 1987a). TA muscle, the largest muscle group in the anterior tibia of the lower limbs innervated by the sciatic nerve, was selected for *in vivo* muscle contractions measurement in our present study. Another group also reported that the twitch and tetanic force of isolated diaphragm from metformin-treated mdx mice were significantly improved (Mantuano et al., 2018). Considering muscle weakness is the major cause of dyskinesia in DMD patients, these results can be considered as a potential therapeutic outcome.

Due to the lack of dystrophin, mdx muscle fibers have repeated degeneration/regeneration processes (Torres and Duchen, 1987; Coulton et al., 1988; McGeachie et al., 1993). Our results revealed that the drug significantly improved TA muscle degeneration, decreased the percentage of muscle damage and increased the percentage of central nuclear muscle fibers and eMyHC+ newly formed muscle fibers (Figure 3), suggesting that more muscle fiber regeneration occurred after metformin treatment. In addition, the lack of dystrophin leads to improper assembly of DGC to maintain the integrity and stability of the muscle fiber membrane structure, and the muscle fibers become fragile and prone to damage (Campbell and Kahl, 1989). We also showed that metformin significantly improved the incomplete muscle fiber membrane of TA muscles and reduced muscle fragility (Figure 4). Our findings add new insights into metformin for the treatment of DMD muscle injury or degeneration. On the other hand, membrane fragility due to dystrophin deficiency causes intracellular Ca^2+^ dysregulation, subsequently results of substantial production of reactive oxygen species (ROS) and mitochondrial dysfunction. While increased oxidative stress results from an unbalance between increased production of ROS and an insufficient antioxidant response will lead to myonecrosis, inflammatory cell infiltration, adipose tissue accumulation and muscle damage (Whitehead et al., 2010; Pal et al., 2014; Pessina et al., 2015). In particular, increasing oxidative stress was observed in muscle biopsies from DMD patients and reducing ROS level has been recently considered to be one of the treatments for DMD (Scholte et al., 1985; Petrillo et al., 2017). Interestingly, previous studies have shown that metformin has antioxidant properties by enhancing the endogenous cell antioxidant capacity (Onaran et al., 2006; Abd-Elsameea et al., 2014; Zeng et al., 2019). Our results also showed that the percentage of total damage area and the percentage of muscle fibrosis in mdx mice decreased after metformin treatment (Figure 3). Future study is needed to determine whether ROS level are also reduced after metformin treated in mdx mice.

The synergy of muscles and nerves regulates muscle strength. NMJ, a synapse between motor neurons and skeletal muscle fibers, is critical for muscle contraction control. Importantly, both human patients and the mouse model for DMD (the mdx mouse) exhibit fragmented NMJ (Kong, 1999; Adams et al., 2000). Studies in mdx mice have revealed presynaptic and postsynaptic abnormalities, nerve terminal discontinuity, as well as related functional changes in neuromuscular transmission and nerve-evoked electromyography (Kong, 1999; Banks et al., 2009; Pratt et al., 2015). Here, we reported for the first time that metformin improved neuromuscular transmission in dystrophic muscles. Meanwhile, we also found that metformin did not improve NMJ structure deficits in mdx mice (Figure 6), which is not hard to understand because of the stable NMJ structure. However, it needs a further deep examination of how metformin improves neuromuscular transmission.

AMP-activated protein kinase is a major cellular energy sensor and regulator for metabolic homeostasis. In skeletal muscle, AMPK responds to the deprivation of cellular energy by increasing ATP production, and usually is activated during exercise (Jørgensen et al., 2006). We observed increased activation of AMPKα in the metformin-treated mdx mice by using anti-phospho-AMPKα antibody (Figure 7). This finding is consistent with a previous study showing that metformin exerts its effects by activating AMPK in the liver and skeletal muscle (Zhou et al., 2001). The activation of AMPK in dystrophic muscles has great significance for the observed beneficial effects of metformin. Recently, the activation of AMPK has been shown to play an important role in maintaining and remodeling skeletal muscle phenotype in DMD disease (Ljubicic and Jasmin, 2013; Dial et al., 2018a). The chronic induction of skeletal muscle AMPK activity is an attractive treatment for DMD. Activating AMPK mitigates the dystrophic phenotypes and, importantly, can apply to all DMD patients (Ljubicic and Jasmin, 2013; Lynch, 2017). Previous studies have also shown that AMPK activators have beneficial effects in mdx mice. For example, AICAR (an AMPK activator) treatment in mdx mice for 4 weeks significantly improved symptoms including increased overall behavioral activity and limb strength (Jahnke et al., 2012). Our results also suggest that metformin activates AMPK in skeletal muscles of mdx mice. Therefore, the AMPK activation observed in metformin-treated mdx mice may be another potential mechanism for improving dystrophic muscle function (Figure 7). Metformin has previously been shown to protect against apoptosis in cardiomyocyte cell, and activating AMPK has been reported to reduce myocardial apoptosis (Morrow et al., 2003; Sasaki et al., 2009). Reducing muscle cell apoptosis in mdx mice might be another possible mechanism of improve muscle function after metformin treatment. However, further investigation on this issue is needed.

The DGC is localized to the muscle membrane and connects the extracellular matrix to the intracellular skeleton, critical for maintaining muscle cells’ structural stability (Davies and Nowak, 2006; Quan and Gao, 2015). The DGC promotes muscle NO production by regulating the activity of AMPK as a mechanical sensor (Garbincius and Michele, 2015). On the other hand, AMPK stimulation induces the expression of Dystrophin-associated protein complex (DAPC) in skeletal muscle, and the decrease of AMPK is related to DAPC dysfunction (Dial et al., 2018b). These findings indicate that AMPK is sufficient (but not necessary) to affect DAPC levels. However, in mdx mice, the relationship between AMPK kinase and DGC still unknown. In our present study, we also found that metformin, as an indirect activator of AMPK, up-regulated the expression of DGC components in mdx mice. However, the interaction between AMPK activation and DGC components to improve muscle functions in mdx mice needs to be further explored. Notably, both protein and mRNA levels of α-dystroglycan, a component of DGC, were increased after metformin treatment (Figure 7). In DGC, dystroglycan spans the sarcolemma and directly interact with subsarcolemmal proteins and extracellular matrix (ECM) components providing a physical connection between the subsarcolemmal cytoskeleton and basement membrane (Henry and Campbell, 1999; Michele and Campbell, 2003; Lapidos et al., 2004). Studies have shown that the lack of dystroglycan can cause embryonic death in mice (Garbincius and Michele, 2015). When dystroglycan is selectively destroyed in mature muscle fibers, it will lead to fiber instability and degeneration (Dial et al., 2018b). Dystroglycan is cleaved into two subunits after translation, α- and β-dystroglycan. α-dystroglycan is attached to the extracellular cell surface through the transmembrane β-dystroglycan subunit (Ibraghimov-Beskrovnaya et al., 1992; Holt et al., 2000). The polysaccharide carbohydrate moieties of α-dystroglycan are necessary for its binding to ECM proteins such as laminin, agrin, and perlecan (Williamson et al., 1997; Davies and Nowak, 2006; Benziane et al., 2012). Here, our results showed that after metformin treatment in mdx mice, the skeletal muscle membrane’s fragility was reduced and the integrity was increased. We hypothesized that these improvements may be resulted from the upregulation of α-dystroglycan on the surface of the muscle membrane.

One of the most notable limitations of the present study was that some protocols used in our study did not follow the international experimental guidelines and standard operating procedures (SOPs) drafted by independent experts engaged in preclinical research of the mdx mouse model^1^. For example, in the test of measuring muscle strength, we did not follow the SOPs due to the limitation of our instrument. Meanwhile, we also have not measured isometric force of isolated mouse skeletal muscles, which may take advantage on accurate evaluation of muscle contractile function. This may cause some of our data to be unable to be compared with the research of other laboratories, limiting the significance of the present study in pre-clinical research.

In summary, our work showed that metformin enhances muscle functions by affecting the integrity of skeletal muscle cell membranes. The underlying mechanism might mainly rely on the activation of AMPK, the increase of muscle cell regeneration and the decrease of muscle fragility. These findings, combined with the results of recent studies, illustrate the potential efficacy of metformin for DMD treatment, and support the beneficial effects of long-term use of metformin in the treatment of DMD patients with weakened muscle functions. Future research should focus on developing treatment methods to solve the dyskinesia caused by muscle weakness to maximize the recovery of the motor function of DMD patients.

## Data Availability Statement

The raw data supporting the conclusions of this article will be made available by the authors, without undue reservation.

## Ethics Statement

The animal study was reviewed and approved by Institutional Animal Care and Use Committee of the Nanchang University.

## Author Contributions

XL designed and directed the project. XD, TH, and JC performed the research experiments. XD, XL, and EF wrote the manuscript. ZY, DR, SZ, and HJ analyzed the data. SW helped with data interpretation and provided instruction. All the authors contributed to the article and approved the submitted version.

## Conflict of Interest

The authors declare that the research was conducted in the absence of any commercial or financial relationships that could be construed as a potential conflict of interest.

## Footnotes

1 https://treat-nmd.org/

## Abbreviations

DMD: Duchenne muscular dystrophy
AMPK: AMP-activated protein kinase
TA: tibialis anterior
EDL: extensor digitorum longus
mEPP: miniature endplate potentials
EPPs: endplate potentials
DGC: dystrophin-glycoprotein complex
T2D: type 2 diabetes
NMJ: neuromuscular junction
CK: creatine kinase
EBD: evans blue dye
HE: hematoxylin and eosin
CLN: centrally located nuclei
NF: neurofilament
SYN: synaptophysin
α-BTX: α-bungarotoxin
ROS: reactive oxygen species.
